# Flowering Plant Microbiomes and Network Interactions Across an Urban Gradient

**DOI:** 10.1111/1462-2920.70089

**Published:** 2025-03-28

**Authors:** Katherine D. Chau, Makaylee K. Crone, Phuong N. Nguyen, Sandra M. Rehan

**Affiliations:** ^1^ Department of Biology York University Toronto Canada

**Keywords:** anthosphere microbiome, bipartite, co‐occurrence networks, floral health, pollinator, urban land use

## Abstract

We used flowers to explore how ephemeral anthosphere microbiomes differ among flowering plant species and along an urban gradient. Here, we sequenced 16S rRNA for bacteria, ITS1 for fungi and rbcL for plant DNA from 10 different plant species sampled to characterise anthosphere microbiomes along an urban gradient and identify important network interactions. Bacterial and fungal flower microbiomes significantly differed in diversity across plant species, especially among Asteraceae and Fabaceae. Across all analyses, four taxa, the bacteria *Pantoea* and *Rosenbergiella* and the fungi *Alternaria* and *Cladosporium* were highly prevalent and contributed to the majority of microbiome composition differences observed between plant species. These four taxa harbour strains or species that may be either pathogenic or beneficial to plants. Across a land use gradient, the plant community bacterial and fungal microbiome was stable and consistent. Flower‐plant networks confirmed all focal flower families in abundance on each sampled flower, with the addition of Paulowniaceae, suggesting that pollinators visiting the focal flowers also visit this plant family. Our findings reveal that anthosphere microbiomes are diverse at the plant community level and encouragingly remain robust against urbanisation.

## Graphical abstract

Analysis of plant anthosphere microbiomes reveals that the bacterial and fungal microbiomes are diverse in the plant community with no effect of urbanisation. Highly prevalent and abundant bacteria and fungi tend to significantly co‐occur. Detections of rbcL in flower‐plant networks identify *Paulownia* sp. as an important plant for urban pollinators.

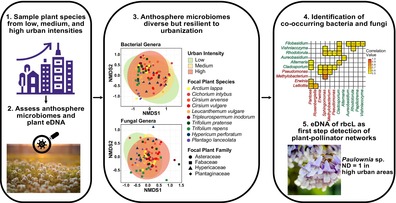

## 1 Introduction

The anthosphere microbiome is an ephemeral microbiome residing on the flower surface. Recent studies have shown that the anthosphere microbiome impacts overall plant health, angiosperm evolution, and pollinator attraction (Shade et al. 2013; Wei and Ashman 2018; Rebolleda‐Gómez et al. 2019; Vannette 2020). Over evolutionary time, angiosperms evolved diverse traits in floral colours, fragrances and shapes to attract animals, as the majority of flowers cannot reproduce without pollination from animals, primarily insects (Ollerton et al. 2011). Bees are designated as the most important pollinator group and help service a EUR 153 billion dollar industry by pollinating 75% of global food crops (Klein et al. 2007; Khalifa et al. 2021). This is due in part to the restrictive diet of bees, which consists of foraging on flowering plants for nectar or pollen that contains all essential nutrients for larval and adult bee development (Vaudo et al. 2015; Crone et al. 2022; Chau and Rehan 2024; Stephen et al. 2024). Despite being an ephemeral feature, flowers contain abundant microbiota and act as hubs of microbe transmission to their pollinators, creating intricate plant–pollinator–microbe networks (Keller et al. 2021).

The composition and abundance of anthosphere microbes play a role in plant disease resistance (Cui et al. 2021; Burgess and Schaeffer 2022) and improve reproduction by altering the floral scent (Peñuelas et al. 2014), taste (Schaeffer et al. 2019), or colour (Hendry et al. 2018) in order to better attract pollinators. For example, the yeast *Metschnikowia* is highly prevalent in plant nectar and secretes volatiles affecting structure and odour, making the nectar more attractive to generalist pollinators (Rering et al. 2017). However, pathogenic microbes may proliferate on flowers, causing various diseases such as fire blight disease by the bacterium *Erwinia amylovora* , causing tissue necrosis (Kong et al. 2021), brown rot blossom blight causing wilting and rotting fruit via the fungus *Monilinia laxa* (Crowley‐Gall et al. 2022) or grey mould on flowers due to the fungus *Botrytis cinerea* (Muñoz et al. 2019). Moreover, high richness of different bacteria was shown to induce avoidance by potential pollinators, thus decreasing overall plant fitness via reduced reproductive rates (Junker et al. 2014). Hence, characterising flower microbiomes is important to elucidate the impacts of certain microbes on the flower as it relates to overall plant health, but it also points to potential disruptions in healthy plant–pollinator–microbe networks which may be caused by environmental stressors.

Climate change and urbanisation are major anthropogenic factors that can alter plant microbiomes, causing dysbiosis and affecting how plants respond to environmental stress. Urbanisation is known to be detrimental to pollinator microbiomes by increasing pathogen exposure (Chau et al. 2023) and decreasing beneficial microbiota (Nguyen and Rehan 2022, 2023). Urbanisation may have minimal impact on anthosphere microbiomes possibly due to its transient nature (Bartlewicz et al. 2016; Gaube et al. 2021; Donald et al. 2022), but studies exploring the direct influence of increasing urban intensities for diverse flower species are woefully limited. Instead, most flower microbiome studies are focused on the rhizosphere or leaf microbiomes which demonstrate highly variable responses of bacterial and fungal microbes to urbanisation (Zhang et al. 2020, 2023; Chen et al. 2021; Yao et al. 2023). Thus, it is necessary to expand research in urban settings to formalise a clearer understanding of the effects of urbanisation on local floral anthosphere communities.

Bipartite network analyses explore the strength and resilience of plant–pollinator or pollinator–microbe networks in an urban framework, which helps characterise key network features most impacted by environmental changes (Dormann and Strauss 2013; Bennett et al. 2018). However, flowers provide an interesting opportunity to utilise environmental DNA metabarcoding to detect other plant DNA as an indirect inference of foraging preferences of pollinators and how their preferences may change along an urban gradient by exploring plant–plant networks. As pollinators visit different flowers, they naturally deposit pollen or other plant DNA on the focal flower that can be sampled through environmental DNA sampling techniques (Newton et al. 2023). Examining both plant–plant networks, alongside plant–microbe networks, provides a holistic approach to approximate pollinator–plant interactions as an initial step that does not require sampling of vulnerable pollinators.

In this study, we characterise the anthosphere microbiome for different flowers sampled along an urbanisation gradient. Here, we expect flower anthosphere microbiomes to differ in diversity and composition between flowers. Secondarily, we expect increasing urbanisation will affect anthosphere microbiome diversity and destabilise plant‐microbe networks. Thirdly, we make inferences on prominent plant–plant interactions using environmental DNA techniques to examine pollination networks along an urbanisation gradient. We predict that environmental DNA can be used to detect other prominent flowers likely visited by local pollinators and to infer plant–pollinator networks along the urban gradient. This is the first study to our knowledge that uses metabarcoding data to infer plant–pollinator relationships and/or threats to local pollination systems through targeted floral assays.

## 2 Experimental Procedures

### 2.1 Sample Sites and Plant Sampling

**FIGURE 1:**
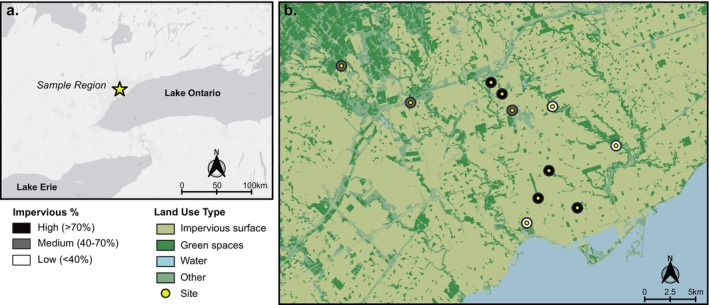
Map of sampling sites. (a) Sample region located in the Greater Toronto Area, Canada. (b) Sample sites (*N* = 11) with the outer buffer circle of 500 m radius coloured by impervious surface % using the Southern Ontario Land Resource Information System (SOLRIS) 3.0. Impervious surface includes transportation and impermeable land; green space consists of pervious surfaces, agricultural land and forest/peat cover; other includes undifferentiated, rock, quarries and land extractions.

Sites were selected to ensure a broad range of impervious surfaces across the Greater Toronto Area sampling region. The proportion of impervious surface (hereafter % impervious) within a 500 m radius of each site was calculated from the Southern Ontario Land Resource Information System (SOLRIS) version 3.0 dataset (Ministry of Natural Resources and Forestry 2019) using QGIS version 3.36.2‐Maidenhead (QGIS.org 2024). Land types associated with SOLRIS class names *transportation* (value 201) and *built‐up area—impervious* (value 203) were included in the % impervious cover calculation. In total, 11 sites were sampled, with 3 sites belonging to low (< 40% impervious surface), 3 sites belonging to medium (40%–70% impervious surface) and 5 sites belonging to high (> 70% impervious surface) urban categories (Figure 1, Table S1).

A total of 10 plant species belonging to four families (Asteraceae, Fabaceae, Hypericeae and Plantaginaceae) and eight genera (*Trifolium*, *Cirsium*, *Cichorium*, *Leucanthemum*, *Actium*, *Tripleurospermum*, *Hypericum* and *Plantago*) were sampled across the sites from June to September 2023. Common plant species across sites were selected. At least three flower heads per plant species from different patches within a site were collected. Flowers of the same species per site were kept together in a plastic bag and stored at −20°C until DNA extraction. In total, 9 biological replicates per plant species were collected for a total of 90 plant samples across 11 sites (12 plant samples from low, 26 from medium and 52 from high % impervious sites).

### 2.2 DNA Extraction

Plant samples from each site were homogenised with a mortar and pestle before sub‐sampling. The mortar and pestle were sanitised with a 10% bleach solution and then rinsed with water to remove any bleach residue between each set of samples. Approximately 0.1 mL of glass beads and one 7‐mm stainless steel bead were kept in Omega Bio‐Tek disruptor tubes and combined with 200‐μL ATL buffer. Homogenised plant species were added to the disrupter tube and further homogenised using a plastic pestle. 30‐μL Proteinase K was added and vortexed in the tube, and the tubes were again homogenised using Disrupter Genie Cell Disrupter Homogeniser for 5 min. Tubes were then incubated at 56°C for approximately 3–4 h with occasional vortexing to ensure dispersion of the sample during incubation. Following incubation, tubes were centrifuged at 12,000 g for 2 min until all cell debris and glass beads were settled. Approximately 150 μL of supernatant was decanted into a new 1.5 mL microcentrifuge tube, and further DNA extraction was done using the QIAGEN DNeasy Blood and Tissue kit, following the manufacturer's protocol.

### 2.3 Sequencing

Paired‐end amplicon sequencing was performed on the Illumina MiSeq system targeting 300 bp regions for the 16S rRNA gene (hereafter 16S) for bacteria, the internal transcribed spacer gene (hereafter ITS) for fungi and the RuBisCo large subunit gene (hereafter rbcL) for plants. The 16S rRNA primers amplified the V5‐V6 fragment using the forward primer 799F‐mod3 (CMGGATTAGATACCCKGG) and the reverse primer 1115R (AGGGTTGCGCTCGTTG) (McFrederick and Rehan 2016). The ITS primers targeted the ITS1F region using the forward primer ITS1F (CTTGGTCATTTAGAGGAAGTAA) and the reverse primer ITS2 (GCTGCGTTCTTCATCGATGC) (McFrederick and Rehan 2019). The rbcL primers used were the forward primer rbcL7 (CTCCTGAMTAYGAAACCAAAGA) and the reverse primer rbcL8 (GTAGCAGCGCCCTTTGTAAC) (McFrederick and Rehan 2016). Library preparation, sequencing and demultiplexing of reads were completed by the Canadian Centre for DNA Barcoding (Guelph, Canada). Raw reads were obtained as FASTQ files and processed for further analyses.

### 2.4 Taxonomy Classification and Decontamination

Raw reads were quality checked and trimmed using the DADA2 pipeline (Callahan et al. 2016) in Qiime2 (Bolyen et al. 2019). Reads were trimmed if Phred quality scores were below 40, and reads were truncated to keep lengths of 250 bp for 16S and 150 bp for rbcL; ITS reads were kept at variable lengths and adaptors trimmed using *cutadapt* version 4.9 (Martin 2011), keeping all reads even with ambiguous bases to retain as many sequences as possible but removing spurious reads shorter than 50 bp following the DADA2 1.8 workflow (https://benjjneb.github.io/dada2/ITS_workflow.html). This resulted in 3314 amplicon sequence variants (ASVs) for bacteria, 2001 ASVs for fungi (read length 58–424 bp) and 377 ASVs for plants. Feature tables for each gene were created to obtain the ASV counts for each sample, and each ASV sequence was used for taxonomy classification. Sequences were BLASTed using blastn (version 2.14.1; Altschul et al. 1990) with the non‐redundant nucleotide *nt* database (last updated on 25 September 2024). Only the top alignment hit was kept for 16S and ITS datasets using –max_hsps 1 and –max_target_seqs 1, but these parameters were removed in order to obtain the top 5 hits for rbcL sequences due to more classification errors with plants at the species level (Martin et al. 2024).

If two or more of the classifications for plant ASVs were identical to the species level, that classification was used. Otherwise, classifications were manually assessed and compared against the USDA PLANTS database (https:// https://plants.usda.gov/) to choose the genus and species that was most appropriate based on sampling region (e.g., if the plant was found in North America). Classifications without ambiguity (e.g., four or more different hits and/or no distribution in North America) were only classified to the most consistent taxonomy level, which was often up to the order or family level only. For bacteria and fungi, we also identified ASVs using pre‐trained feature classifiers in QIIME2 based on the SILVA database (version 138, 99% sequence similarity OTUs full‐length sequences, Robeson 2nd et al. 2021) for bacterial reads and the UNITE database (version 25.07.2023, all eukaryotes, Abarenkov et al. 2024) for fungal reads. Classifications based on BLAST were prioritised due to deeper taxonomy classification, but ambiguous or unknown ASVs were then corroborated with the SILVA and UNITE databases to obtain as complete a dataset for bacteria and fungi, respectively. Unknown genera and families were labelled as ‘Unknown.N’, where *N* is a number to indicate a potentially unique genus or family. Due to a higher degree of uncertainty at the species level, all datasets are analysed to the family and genus level only to reduce ambiguity. Full lineage for all classifications was extracted using *efetch* from NCBI Entrez Direct E‐Utilities (version 15.3) (https://www.ncbi.nlm.nih.gov/books/NBK25499/) using the blast taxonomy ids (i.e., ‘taxids’).

For the bacterial, fungal and plant datasets, the ASV feature table and classifications were further cleaned by identifying contaminants in blank samples using the PHYLOSEQ package (McMurdie and Holmes 2013) in R (version 4.2.1) (R Core Team 2021), followed by removal of contaminant ASVs with the DECONTAM package (Davis et al. 2017). Furthermore, ASVs were removed if there was no classification to kingdom level (i.e., Kingdom was ‘N/A’), or if the ASV was an incorrect domain. For bacteria, ASVs matching to kingdom ‘Eukaryota’ were removed, along with intercellular bacterial genera contaminants *Sodalis* and *Wolbachia* (Graystock et al. 2017). For fungi, ASVs matching to phylum ‘Arthropoda’ and ‘Streptophyta’ were removed. For plants, ASVs matching to phylum ‘Arthropoda’ were removed. This resulted in final datasets of 3051 ASVs for bacteria across 88 samples, 1876 ASVs for fungi across 90 samples and 354 ASVs for plants across 90 plant samples. Finally, we used a relative abundance threshold of 0.01%, converting ASV abundances in a sample below this threshold to 0.

### 2.5 Diversity Analyses

For all analyses and results discussing relationships between sampled plant species and rbcL plant DNA, we refer to the samples as *flowers* and rbcL data as *plant* data. All analyses using bacteria or a combined bacteria and fungi (hereafter bacteria+fungi) dataset were dropped to 88 plant samples due to the removal of samples MKC202 and MKC254 as there was a lack of bacterial reads in these samples; both samples were from *Cichorium intybus* plant samples.

Diversity analyses were done on the complete dataset with all samples, and on separate datasets for each of the 10 sampled plant species and two plant genera that had more than one plant species representative (e.g., *Cirsium* and *Trifolium*). To test for significant differences in the diversity of bacteria, fungi and plant taxa communities between plant species and the two genera, we used the Shannon‐Wiener alpha diversity index, which reflects how rich and even the taxa community is between samples, from the R package VEGAN (Feranchuk et al. 2018; Oksanen et al. 2020). We also tested if alpha diversity for each flower grouping differed across % impervious surfaces using both continuous values and categorical values (e.g., low, medium, high). The Shapiro–Wilks test in R using the *shapiro.test* function was first used to assess if alpha diversity indices were normally distributed, followed by ANOVA testing using the *aov* function, and post hoc testing with Tukey's honest significant difference (HSD) via *HSD.test*. If the data was not normally distributed, instead, the Kruskal–Wallis test via *kruskal.test* was implemented for non‐parametric testing of alpha diversity for plants, followed by Dunn post hoc testing from the R package FSA (Ogle et al. 2023) via *dunnTEST*.

Next, we used the R package VEGAN to calculate Bray‐Curtis dissimilarities on the microbiome datasets with abundances transformed using the *Hellinger* method in the *decostand* function. Next, PERMANOVA analysis via *adonis2* from the VEGAN package was used to test for significant differences in composition across the flower groups (i.e., plant species, genera and families). PERMANOVA was also used to test for the impact of urbanisation across all plant samples using both continuous and binned % impervious values. If PERMANOVA was significant, the *betadisper* method was used to ensure homogeneity of variances. If *betadisper* was not significant (i.e., PERMANOVA is *not* violated), Tukey HSD using the *TukeyHSD* method was implemented to identify key flower group pairs. For the plant datasets, PERMANOVA was violated due to significant *betadisper* tests. We visualised Bray‐Curtis dissimilarities using non‐metric multidimensional scaling (NMDS) plots by first running *metaMDS* from the VEGAN package in R for each flower group, as well as continuous and binned % impervious values. We then separated the bacteria, fungi and plant datasets by plant species, family or genus in order to assess how alpha and beta diversity differ in specific floral groupings along an urbanisation gradient. We repeated the alpha and beta diversity calculations using rarefied datasets by rarefying to an even depth of 5000 reads per sample, using the *rarefy_even_depth* function from the PHYLOSEQ package.

Next, to assess top microbiota contributing to flower microbiome composition differences, we ran similarity percentage tests (SIMPER) using Bray–Curtis dissimilarity in the PAST (Hammer et al. 2001) program for bacteria and fungi. The top 10 taxa based on their contribution values were identified for each flower at the species and genus levels as well as urbanisation level (high, medium and low). As a second method to detect significantly co‐occurring taxa, we used the co‐occurrence network inference (CoNet) from the R package (CONETINR; Faust and Raes 2016), focusing on the top taxa families and genera identified across all SIMPER analyses. In order to detect significantly co‐occurring bacteria and fungi, we combined the top bacteria and fungi identified from SIMPER into one family‐level or genus‐level dataset and normalised the reads using cumulative sum scaling which addresses compositional biases due to sequencing depth differences (Xia 2023) via the METAGENOMESEQ package (Paulson et al. 2013). CoNet networks were created using Pearson, Bray‐Curtis, Spearman and Kullback–Leibler edge scores, 100 iterations, and then Benjamini‐Hochberg correction for multiple testing. Correlations with significant p‐values above and below the diagonal for a taxa pair were deemed significant correlating taxa.

### 2.6 Taxa Overrepresentation and Co‐Expression

The R package DESEQ2 (Love et al. 2014) was used for negative binomial distribution analysis (NBDA) to identify microbiota (bacteria or fungi) that were significantly overabundant between plant species and genera as well as among % impervious bins. Prior to this, differences in sequencing depth and variability in abundances were accounted for by estimating size factors using the *estimateSizeFactors* (type = ‘poscounts’ for zero inflated data) and *estimateDispersions* methods implemented in DESEQ2. For flower comparisons, the group of interest was compared against all other groups (e.g., *Arctium lappa* versus all other plant species). Next, we incorporated random forest classification (RFC) testing from the RANDOMFOREST package (Liaw and Wiener 2002) to test if bacterial or fungal datasets can predict plant species, genus, or % impervious level. The RFCs were trained across nine training set sizes ranging from 10%–90% (increasing by 10%) and repeated 10 times for an average accuracy percent. Before each RFC we ran the *tuneRF* method to obtain an optimal mtry value which determines the minimum number of taxa to select for minimal error, for 5000 runs (ntree = 5000). Next, the CARET package (Kuhn 2008) was used to create a confusion matrix that summarises overall RFC performance. This was followed by extracting the top 10 important taxa that influenced the RFC accuracy for each seed (10 runs) for the 80% training dataset size, using the *measure_importance* method from the RANDOMFORESTEXPLAINER package (Paluszynska et al. 2020). Taxa that showed up as an important feature for all 10 seed trials are considered highly influential for the RFC model.

Plant DNA from other flowers may be deposited on our focal plant species by pollinators; hence, we analyse plant–plant interactions (hereafter ‘flower–plant interactions’) to indicate sampled plant (species interacting with detected plants via rbcL sequencing) alongside ‘flower–microbe’(i.e., either flower–bacteria or flower–fungi) interactions. As a control, we expect the majority of the rbcL reads to belong to our focal plant species' families or genera. The bacteria and fungi datasets were assessed using the BIPARTITE R package (Dormann et al. 2009). Network interaction indices connectance, interaction evenness (IE), weighted nestedness (WN), robustness of flowers and robustness of taxa (i.e., bacteria, fungi, plants) were extracted using the *networklevel* function. Species interaction metrics were obtained using the *specieslevel* function to obtain degree and normalised degree (ND) to determine the most interacted microbiome and plant taxa across the entire dataset, and for each urbanisation category. Networks using all sites were visualised using the *plotweb* function and only included taxa with total abundances over 1000 and that have prevalence in at least 75% of sampled flowers. The datasets were further divided into urbanisation intensity levels to only assess flower‐taxa or flower‐plant interactions that occurred at each urbanisation intensity.

Results for all statistical analyses were done at both the family‐level and genus‐level data but were largely similar and thus, results and discussion will primarily focus on genus‐level results, unless mentioned otherwise. Majority of family‐level results are presented in the Supporting Information.

## 3 Results

### 3.1 Sequencing and Samples

Metabarcoding using Illumina MiSeq resulted in a total of 3,470,250 reads for 16S with an average of 11,341 reads per sample, 5,588,427 reads for ITS with an average of 18,263 reads per sample and 15,777,833 reads for rbcL with an average of 51,562 reads per sample. After cleaning, final datasets consisted of 3051 ASVs for 16S across 88 samples, 1876 ASVs for ITS across 90 samples and 354 ASVs for rbcL across 90 samples for further analysis (Tables S2–S4). After merging ASVs by genus and using a relative threshold abundance of 0.01%, this corresponded to 407 bacterial genera (2 families), 303 fungal genera (176 families) and 69 plant genera (34 families).

### 3.2 Microbiome Diversity Across the Flower Community

**FIGURE 2:**
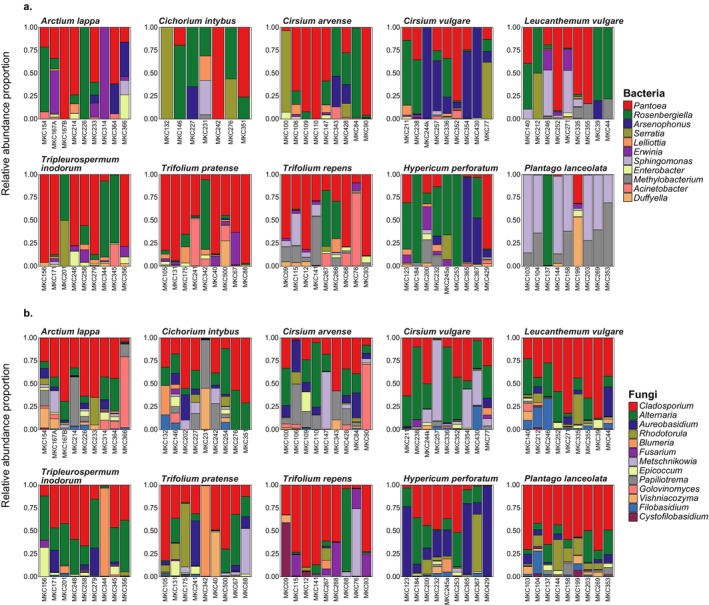
Top (a) bacterial and (b) fungal genera distribution for each plant species based on relative abundance. The top taxa are identified if they were prevalent in ≥ 25% of all samples and have over 1% total relative abundance across the entire dataset.

**FIGURE 3:**
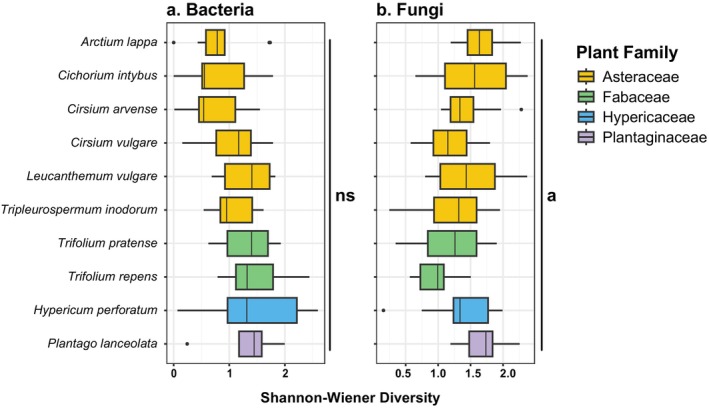
Shannon–Wiener alpha diversity across plant species for (a) bacteria and (b) fungi genus‐level data. Boxplot bars are coloured by plant species' family. NS = non‐significant result, whereas ‘a’ indicates that Tukey HSD did not detect significantly different pairs, but ANOVA was significant. Plots are based on the unrarefied dataset (Table S5).

**FIGURE 4:**
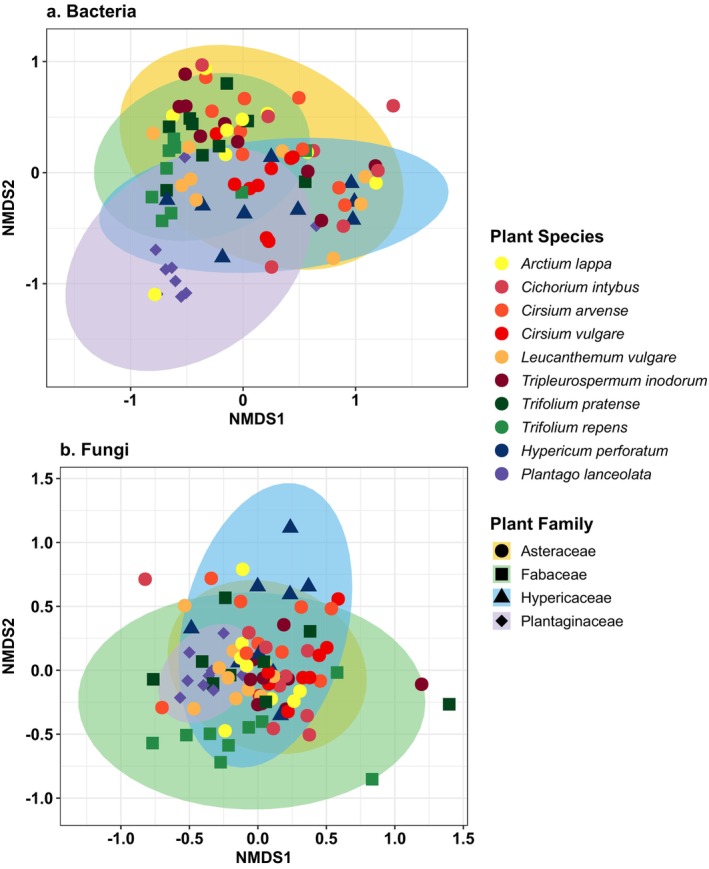
Non‐metric dimensional scaling (NMDS) plots for (a) bacterial and (b) fungal genera across plant species. Ellipses and shapes are coloured or shaped by plant family.

**FIGURE 5:**
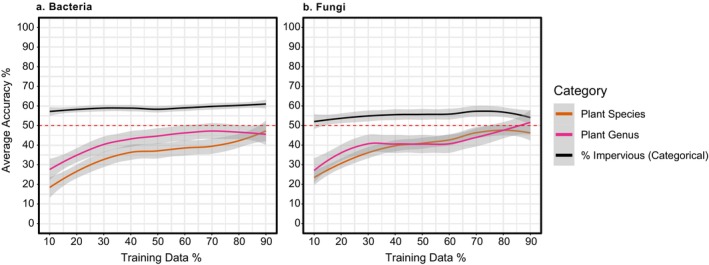
Overall average accuracy for each random forest classification (RFC) model for (a) bacterial and (b) fungal genera. Each RFC tested the predicative ability of using the microbial genus‐level abundance dataset to predict plant species, plant genus or urban intensity (low, medium, high). Red dashed line indicates a 50% average accuracy performance. Error bars generated using loess smoothing curve method. Full results shown in Table S11.

Top bacterial genera with over 1% relative abundance and over 25% prevalence across the entire bacterial dataset consisted of 11 genera (Figure 2a). *Pantoea* was the most abundant bacterial genus, making up 35% of the dataset with 85% prevalence, followed by *Rosenbergiella* (26% composition, 77% prevalence). These two bacterial genera contributed to 50% of the differences in bacterial composition across plant species (Table S5). These genera were dominant in almost all plant species except for *Hypericum perforatum* and *Plantago lanceolata* . Bacterial genera richness was similar across all plant species (Figure 3a), whereas family richness significantly differed across plant species in both the unrarefied (ANOVA F = 2.7, df = 9, *p* = 0.0083, Figure S1a, Table S6) and rarefied datasets (ANOVA F = 3.1, df = 8, *p* = 0.0061, Table S7). *Plantago lanceolata* significantly had higher bacterial family richness compared to the aster *Cirsium arvense* , and it was the only plant species with variable bacterial composition (Figure 4a) with significant overrepresentation by the bacterium *Sphingomonas* (Table S8). Bacterial family and genus beta diversity significantly differed across plant species in both the unrarefied (PERMANOVA *F* = 3.9, df = 9, *p* = 0.001, Table S9) and rarefied datasets (PERMANOVA *F* = 3.9, df = 9, *p* = 0.001, Table S10), but pairwise differences were not detected. Although our RFC models showed that bacterial composition was only able to weakly predict plant species and plant genera as average accuracy did not exceed 50% at any training data set size (Figure 5a), the models consistently identified top bacterial genera as important predictors and included top genera *Pantoea*, *Rosenbergiella*, *Arsenophonus*, *Acinetobacter*, *Sphingomonas*, *Pseudomonas* and *Methylobacterium* (Table S11).

**FIGURE 6:**
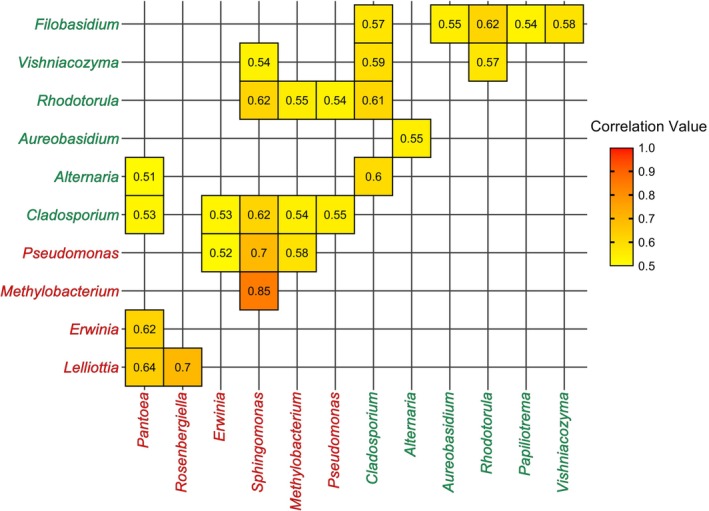
Correlation network (CoNet) values for positive correlations between taxa that are ≥ 0.5 and have significant *p*‐values in the upper and lower triangles (see Table S14). Fungal genera are coloured in green and bacterial genera are coloured in red.

There were 13 fungal genera that were highly prevalent and abundant and made up similar compositions across the plant species (Figure 2b). *Cladosporium* dominated all plant species as it occurred in 100% of samples and made up 36% of the fungal genus. *Cladosporium*, followed by *Alternaria* (20% composition, 98% prevalence) and *Aureobasidium* (10% composition, 90% prevalence) contributed to 57% of the differences in fungal composition observed across plant species (Table S5). Fungal alpha diversity only significantly differed across plant species at the family level using the unrarefied dataset (ANOVA F = 2.5, df = 9, *p* = 0.013, Table S6), whereas fungal genus diversity was similar across plant species (Tables S6 and S7). Fungal genera beta diversity significantly differed across plant species (Tables S9 and S10); however, fungal microbial community clustering was less evident at the genus (Figure 4b) or family level (Figure S2b). The top three fungal contributors, along with *Metschnikowia, Papiliotrema* and *Rhodotorula*, were identified as key predictors for plant species and plant genera classification, although, similar to bacteria, fungal microbiome composition was unable to strongly predict plant species or plant genus (Figure 5b, Table S11). Significant bacteria–fungi correlations detected across all flower samples were consistently between bacteria and fungi previously identified as top taxa and include the fungi *Cladosporium*, *Alternaria* and *Rhodotorula* with several top bacteria such as *Pantoea*, *Sphingomonas*, *Pseudomonas* and *Methylobacterium* (Figure 6, Table S14). Interestingly, the top fungus *Aureobasidium* did not show significant and high correlation with other bacteria, but it did strongly correlate with other fungi such as *Alternaria* or *Filobasidium*.

### 3.3 Microbiome Composition Across an Urban Gradient

Overall, urbanisation had no significant impact on bacterial or fungal richness (Tables S6 and S7) nor on bacterial beta diversity (Tables S9 and S10). The same two bacteria identified as top contributors to bacterial dissimilarity across plant species, *Pantoea* and *Rosenbergiella*, remained the top two genera contributing to 50% of the bacterial dissimilarity across the urban gradient (Table S5). Similarly, *Cladosporium*, *Alternaria* and *Aureobasidium* remained the top three genera contributing to over 50% of the fungal dissimilarity along the urban gradient (Table S5). Only the bacterium *Shigella* was affected by urbanisation, with significant overrepresentation in medium and high urban intensities (Table S8). Unrarefied fungal beta diversity at the family level was the only microbial dataset significantly affected by urbanisation, although pairwise comparisons between urban intensities were non‐significant (Table S9). Only the fungus *Filobasidium* was affected by urbanisation and was overrepresented in medium urban intensities (Table S8). Neither the bacterial nor the fungal microbiome datasets showed predictive power regarding urban intensity (Figure 5, Table S11).

**FIGURE 7:**
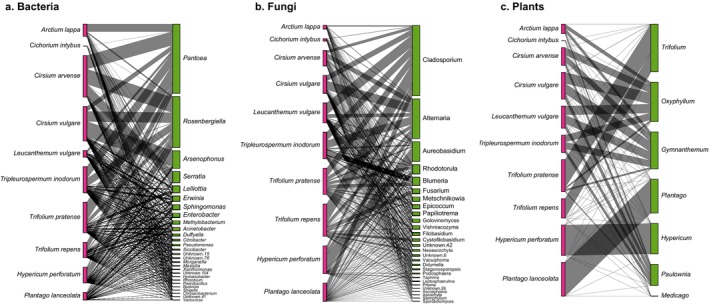
Flowering plant species (left) to taxa (right) network for (a) bacterial genera, (b) fungal genera and (c) plant genera. Networks only include taxa genera that interact with at least 75% interaction with flowers and have a total abundance greater than 1000 reads.

Furthermore, flower–microbe networks did not reveal any dramatic shifts in network stability and suggest that flower–bacteria and flower–fungi networks are generally stable despite increasing urbanisation (Table S12). Interestingly, high urban sites showed highest Shannon diversity for bacteria and fungi, as well as highest IE for both microbial datasets (Table S12). Comparatively, flower–fungi networks always had higher connectance than flower–bacteria networks at each urban intensity, whereas flower–bacteria networks have higher WN and are more nested and structured than flower–fungi networks (Figure 7a). *Pantoea* and *Rosenbergiella* remained as top interacting species (ND = 1), but only in medium and high urban intensities, whereas there were multiple highly interacting fungi present in all urban intensities (Table S13). Bacteria–bacteria and fungi–fungi correlations remained fairly similar for each urban intensity, but bacteria–fungi correlations were more variable. In all intensities, there were significant correlations with the bacteria *Sphingomonas* but with different fungi depending on the landscape (Table S14). For example, in low intensities, *Sphingomonas* correlated only with *Rhodotorula*. In medium intensities, it correlated only with *Vishniacozyma*. But in high intensities, it correlated with *Cladosporium*, *Rhodotorula* and *Filobasidium*. *Pantoea* only had significant correlations with fungi in medium and high urban intensities, whereas low intensities uniquely had correlations with fungi and the bacteria *Lelliottia*.

### 3.4 Flower–Plant Networks

Alpha diversity of plant detected species was significantly different across sampled plant species (Table S6). There were several differences in plant richness between plant species, notably with higher plant richness between Asteraceae focal flowers and *Trifolium* spp. or *Plantago lanceolata* (Figure S3, Tables S6 and S7). Plant beta diversity significantly differed across the local plant community, but there were no significant differences in pairwise plant species comparisons (Tables S9 and S10). Rather, plant diversity formed clear clusters based on focal plant family (Figure S4).

Furthermore, plant richness was significantly greater in medium urban intensities when using the rarefied dataset (Table S7). Plant composition also significantly differed across plant species but remained unchanged across the urban gradient (Figure S5, Tables S9 and S10). Flower–plant networks remained consistent across urban intensities, although low urban intensities are more specialised due to the lowest connectance and greatest weighted nestedness compared to medium and high urban intensities (Table S12). Among the top interacting plant families, the four focal flower families interacted with all other detected plants (i.e., ND = 1) in medium and high urban intensities, whereas *Hypericaceae* did not have complete interactions in low urban intensities (Table S13). Additionally, Paulowniaceae (a family of hardwood trees and was not one of the focal flower families) also had complete interactions with all other plants in high urban intensities.

## 4 Discussion

This study aimed to characterise the anthosphere microbiome for our plant community sampled across an urbanisation gradient, as well as determine important flower–plant interactions as a proxy for plant–pollinator relationships in this study region. While microbial alpha and beta diversity measures significantly differed across plant species, urbanisation had little impact on flower anthosphere microbiomes. Our plant community microbiome compositions were heavily influenced by the abundance of two bacteria, *Pantoea* and *Rosenbergiella* and three fungi, *Cladosporium*, *Alternaria* and *Aureobasidium*. Furthermore, our data suggest that flower–bacteria networks across the urban landscape are more structured, likely due to more specialised bacteria on certain flowers, whereas flower–fungi networks are more balanced due to greater evenness of interacting fungi and flowers. Plant diversity via rbcL detections also differed in richness and beta diversity across focal flowers but remained similar across an urban gradient, suggesting that plant–pollinator networks are fairly unchanged across the urban landscape. However, we note that in high urban systems, pollinators are potentially visiting plants from Paulowniaceae among our focal plant families, as this was the only non‐focal flower family identified that interacted with all studied flowers.

### 4.1 Local Plant Community Anthosphere Microbiomes Are Dominated by a Few Taxa

We found that microbiome richness and composition significantly differed across plant species at the community level but showed almost no differences in pairwise plant species comparisons, which disagrees with our expectation that anthosphere microbiomes differ between plant species. This may be attributed to just a few taxa being identified as highly abundant and prevalent in our plant species. Several studies have found that floral microbiomes are dominated by a few microbes, mainly bacteria belonging to the phyla Proteobacteria (syn. Pseudomonadota) (Manirajan et al. 2018; Steven et al. 2018; Cui et al. 2021; Qian et al. 2021) and fungi belonging to phyla Ascomycota (Manirajan et al. 2018; Qian et al. 2021). Similarly, we found overrepresentation of a few bacteria, namely, *Pantoea* (Pseudomonadota) and *Rosenbergiella* (Erwiniaceae), as well as fungi *Cladosporium* (Ascomycota: Cladosporiaceae), *Alternaria* (Ascomycota: Pleosporaceae) and *Aureobasidium* (Ascomycota: Saccotheciaceae). These genera are common in plant microbiomes (Zhou et al. 2021; Brandl et al. 2023) and were found to co‐occur in our data, which may be of concern as some of these genera have known plant pathogenic species. Certain *Pantoea* strains (e.g., *P. agglomerans* or *P. ananantis*) and *Alternaria* (e.g., *A. alternaria*) cause leaf spot disease (Logrieco et al. 2009; Tho et al. 2015; Yazdani et al. 2017), while *Cladosporium* (e.g., *Cladosporium cladosporioides*) causes blight (Nam et al. 2015; Mukhtar et al. 2021). These three taxa have been found as common constituents in the microbiomes of diseased plants (Xiang et al. 2022; Feng et al. 2023). Additionally, the bacterial genus *Rosenbergiella* is quite common in insect‐pollinated plants, and certain strains such as *R. nectarea* encode virulent genes that may potentially attack plant tissue (Graystock et al. 2017; Manirajan et al. 2018; Laviad‐Shitrit et al. 2020).

However, the top identified genera may instead be comprised of beneficial species. For example, *Aureobasidium* (e.g., *A. pullulans*) is commonly used to control plant pathogens and was highly prevalent in our study (Bozoudi and Tsaltas 2018). *Rosenbergiella nectarea* (detected in our study) may instead be involved with plant volatile production in order to attract pollinators or deter herbivores (Laviad‐Shitrit et al. 2020). Similarly, the presence of *Pantoea*, *Cladosporium* and *Alternaria* may also be indicative of healthy plants as they commonly co‐occur in high abundance in healthy plants (Feng et al. 2023). However, high detections of *Cladosporium* and *Alternaria* are potentially concerning for our study as the dominant identified ASVs belonged to *C. cladosporioides* or *A. tenuissima* and *A. infectoria*, which have plant pathogenic traits (Silva et al. 2014; Nam et al. 2015; Mukhtar et al. 2021; Ahmad et al. 2024). These microbes might also promote plant growth via their alternative properties, such as the presence of antifungal capabilities of *C. cladosporioides* (Torres et al. 2017), or entomotoxic metabolites to deter insects, as present in *Alternaria* spp., including *A. tenuissima* and *A. infectoria* (Salimova et al. 2021). Highest abundances of *Pantoea* ASVs were also identified as pathogenic, belonging to *P. agglomerans* and *P. ananatis* , and were often found in highest abundance in high urban sites. This poses a risk to plants by decreasing pollinator visitation rates in urban environments, as high levels of *P. ananatis* in sugar solutions were found to deter honey bees (Scheiner et al. 2020). Further monitoring of local flower microbiomes and their physiology will be needed to ascertain if the *Pantoea* strains are indeed negatively impacting the overall plant health, as they may only impact certain plant species due to species‐specific flower resistance. As an example, *P. agglomerans* was found in high abundance in healthy apple blossom flowers (Kong et al. 2021). Subsequently, other strains of *Pantoea* (e.g., *Pantoea* sp. MSR2) were found to promote plant growth or act as biological control agents (Nascimento et al. 2020) and warrant further sequencing of bacterial communities in the local flora to verify the unclassified *Pantoea* strains to the species level. This approach may alleviate concerns for plant health in our study system, as it may be that the flowers harbour a diverse mix of beneficial bacterial strains alongside abundant levels of advantageous fungal isolates that are proven to promote plant growth.

### 4.2 Flower Anthosphere Microbiomes Are Resilient to Urbanisation

Our expectations that urbanisation will change flower anthosphere microbiome diversity and destabilise flower–microbe networks were not met. Overall, increasing urban intensity had little impact on bacterial and fungal communities in our focal plant species, and flower–microbe networks demonstrated similar network metrics across the urban landscape. Plant–pollinator networks studied in cities in eastern North America appear to be less resilient (connectance values < 0.1) (Mathiasson and Rehan 2020; Ayers and Rehan 2023), unlike networks with microbes. Although we did not test for flower–pollinator interactions, we observed higher values of flower–microbe connectance, comparable to pollen provision–microbe networks conducted in the same study region (Nguyen and Rehan 2022). In general, bacterial richness is often higher in flower and pollen microbiomes compared to fungi (Manirajan et al. 2018; Qian et al. 2021). We observed a more nested flower–bacteria network as opposed to a more balanced flower–fungi network in our study system. This suggests that flower–bacteria associations may be more specialised in our system compared to fungi. Increased specialised flower–bacteria interactions were also present in another study (Feng et al. 2019), whereas another study found flower–fungi interactions were more specialised compared to bacteria, although differences may be due to exploration of the plant roots and rhizosphere instead of the anthosphere as in our study (Maurice et al. 2024).

While highly urbanised sites tend to promote the spread of pathogenic microbiota in bees (Youngsteadt et al. 2015; Chau et al. 2023), it is still unclear but not difficult to suppose that pathogen load would also increase for plants in urban settings via plant–pollinator vector transmission. In our study, high urban intensities had many co‐occurring taxa with several correlations between bacterial and fungal genera with common pathogenic strains suggesting that load and exposure to potential pathogens increase with urbanisation. However, further studies monitoring the prevalence of known plant pathogens and assessing flower physiology for disease symptoms are warranted to conclude if our plant community is afflicted with diseases in urban areas.

### 4.3 Flower–Plant Interactions

As pollinators visit a variety of plants, we infer top plants important for local pollinators indirectly via flower–plant interactions. The flower–plant networks we observed may indirectly be indicative of plant–pollinator networks across an urban gradient, as we observed greater flower–plant interactions via greater connectance in low urban sites alluding to more frequent pollinator visitations. This could be due to limited foraging spaces in cities via limited dispersal corridors (Chau et al. 2023), despite the overall greater plant diversity and pollinator richness found in cities (Wilson and Jamieson 2019; Rahimi et al. 2022), as we did also observe greater plant diversity in high urban sites. Flower–plant networks confirmed the detection of the focal flora across sampled flowers, but, as per our expectation, also identified other important floral associations for our local pollinators. Flower–plant networks identified Paulowniaceae as a potentially highly visited flower family in high urban intensities. This is an insect‐pollinated family of hardwood trees that is native to China but has spread globally as an ornamental tree (Schneiderová and Šmejkal 2015; Shankar and Abrol 2016), and releases glyceride secretions on its flowers and leaves which may attract and/or reward pollinators (Kobayashi et al. 2008). We use these findings as a first step to obtaining a broad view of other plant families or genera that may be important for urban pollinators and be relevant for conservation focus. As such, future studies focusing on preferred foraging plant species by local pollinators may want to include Paulowniaceae flowers as a focal plant family for both pollinator nutritional studies and plant–pollinator networks.

## 5 Conclusion

In conclusion, this study characterised the community anthosphere microbiome for 10 plant species and found that microbiome richness and composition do differ within the community, but overall, bacterial and fungal diversity is similar between plant species. This is likely due to the high dominance of just a few microbes that were highly abundant and prevalent in our study. Promisingly, urbanisation appeared to have minimal effects on flower anthosphere microbiomes. This suggests that anthosphere microbiomes, possibly due to their transient nature, are likely to remain hardy and resilient with changing urban land use. Our study also isolated five highly abundant and significantly co‐occurring taxa, the bacteria *Pantoea* and *Rosenbergiella*, and three fungi *Cladosporium, Alternaria* and *Aureobasidium* as important taxa that contributed the most to differences in microbial diversity between plant species and urban intensities. Future monitoring and detailed functional experiments of these microbes are needed to determine if they pose a risk or benefit to the flowers, and subsequently the pollinators they support. Flower–plant interaction networks identified interesting interactions possibly indicative of local pollinator foraging behaviour and suggest a role of environmental DNA monitoring to not only document microbial ecology across landscapes but also trophic interactions. These data provide an important first step towards more holistic urban green space initiatives and plant–pollinator health recommendations.

## Author Contributions

**Katherine D. Chau:** writing – original draft, methodology, visualization, formal analysis, data curation. **Makaylee K. Crone:** investigation, methodology, writing – review and editing, data curation. **Phuong N. Nguyen:** investigation, methodology, validation, writing – review and editing. **Sandra M. Rehan:** conceptualization, investigation, funding acquisition, writing – review and editing, project administration, supervision, resources.

## Conflicts of Interest

The authors declare no conflicts of interest.

## Supporting information


**Figure S1.** Shannon–Wiener alpha diversity across plant species for (a) bacteria and (b) fungi family‐level data. Boxplot bars are coloured by plant family. Significance letters based on Tukey HSD post hoc test. Plots are based on the unrarefied dataset (Table S5).
**Figure S2.** Non‐metric dimensional scaling (NMDS) plots for (a) bacterial and (b) fungal families across plant species. Ellipses and shapes are coloured or shaped by plant family, respectively.
**Figure S3.** Shannon–Wiener alpha diversity across plant species for (a) plant family‐level abundance data and (b) plant genus‐level abundance data based on rbcL relative abundance. Boxplot bars are coloured by sample plant family. Significance letters are based on post hoc Dunn's test.
**Figure S4.** Non‐metric dimensional scaling (NMDS) plots for (a) plant data at the family‐level and (b) plant data at the genus‐level across sample plant species. Ellipses and shapes are coloured or shaped by plant family, respectively.

**Table S1.** Sample collection.
**Table S2.** Bacterial read counts (16S).
**Table S3.** Fungi read counts (ITS1).
**Table S4.** Plant read counts (RBCL).
**Table S5.** SIMPER.
**Table S6.** Alpha diversity across all flowers (UNRAREFIED).
**Table S7.** Alpha diversity across all flowers (RAREFIED).
**Table S8.** Overrepresented bacterial and fungal taxa determined from R package DESeq2 for flower species, flower genera and urban intensity categories using the relative abundance ≥. 0.01% datasets. Comparisons are done against flower group of interest versus all other groups.
**Table S9.** Beta diversity across all flowers (UNRAREFIED).
**Table S10.** Beta diversity across all flowers (RAREFIED).
**Table S11.** Random forests.
**Table S12.** Bipartite *Networklevel*.
**Table S13.** Bipartite *Specieslevel*.
**Table S14.** Correlating taxa.

## Data Availability

Data to support the findings in this study are available in NCBI in BioProject PRJNA1120066.
