# Supplementary material for: Flowering Plant Microbiomes and Network Interactions Across an Urban Gradient
Source: Environ Microbiol. 2025 Mar 28;27(4):e70089. doi: 10.1111/1462-2920.70089 (PMC11950910; doi:10.1111/1462-2920.70089)
Supplement: Supplementary file 1 — Figure S1. Shannon–Wiener alpha diversity across plant species for (a) bacteria and (b) fungi family‐level data. Boxplot bars are coloured by plant family. Significance letters based on Tukey HSD post hoc test. Plots are based on the unrarefied dataset (Table S5). Figure S2. Non‐metric dimensional scaling (NMDS) plots for (a) bacterial and (b) fungal families across plant species. Ellipses and shapes are coloured or shaped by plant family, respectively. Figure S3. Shannon–Wiener alpha diversity across plant species for (a) plant family‐level abundance data and (b) plant genus‐level abundance data based on rbcL relative abundance. Boxplot bars are coloured by sample plant family. Significance letters are based on post hoc Dunn's test. Figure S4. Non‐metric dimensional scaling (NMDS) plots for (a) plant data at the family‐level and (b) plant data at the genus‐level across sample plant species. Ellipses and shapes are coloured or shaped by plant family, respectively. [file EMI-27-e70089-s001.docx]

**Flowering plant microbiomes and network interactions**

**across an urban gradient**

**Supplementary Figures**


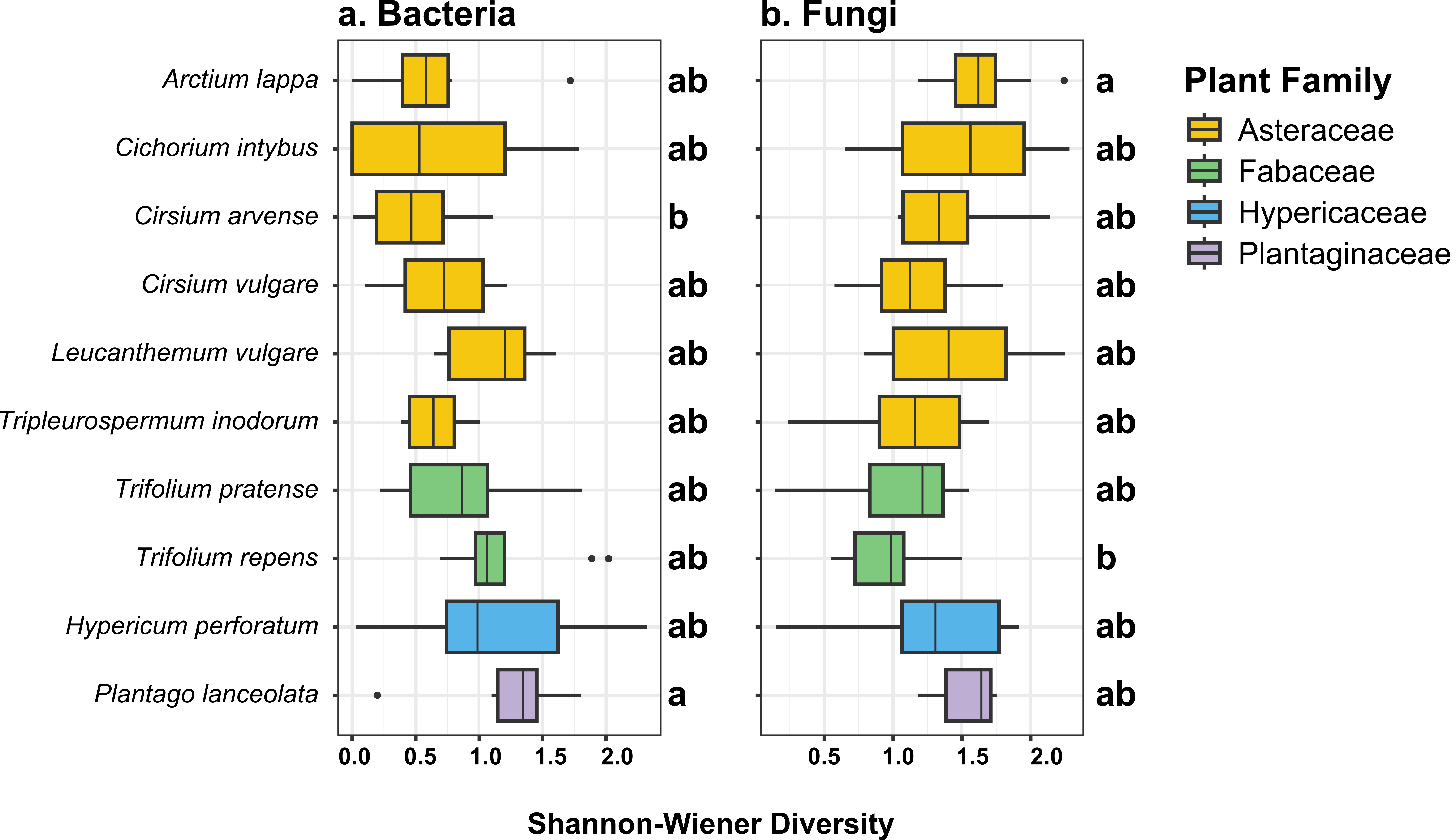


**Figure S1.** Shannon-Wiener alpha diversity across plant species for (a) bacteria and (b) fungi family-level data. Boxplot bars are coloured by plant family. Significance letters based on Tukey HSD post hoc test. Plots are based on the unrarefied dataset (**Table S5**).

**
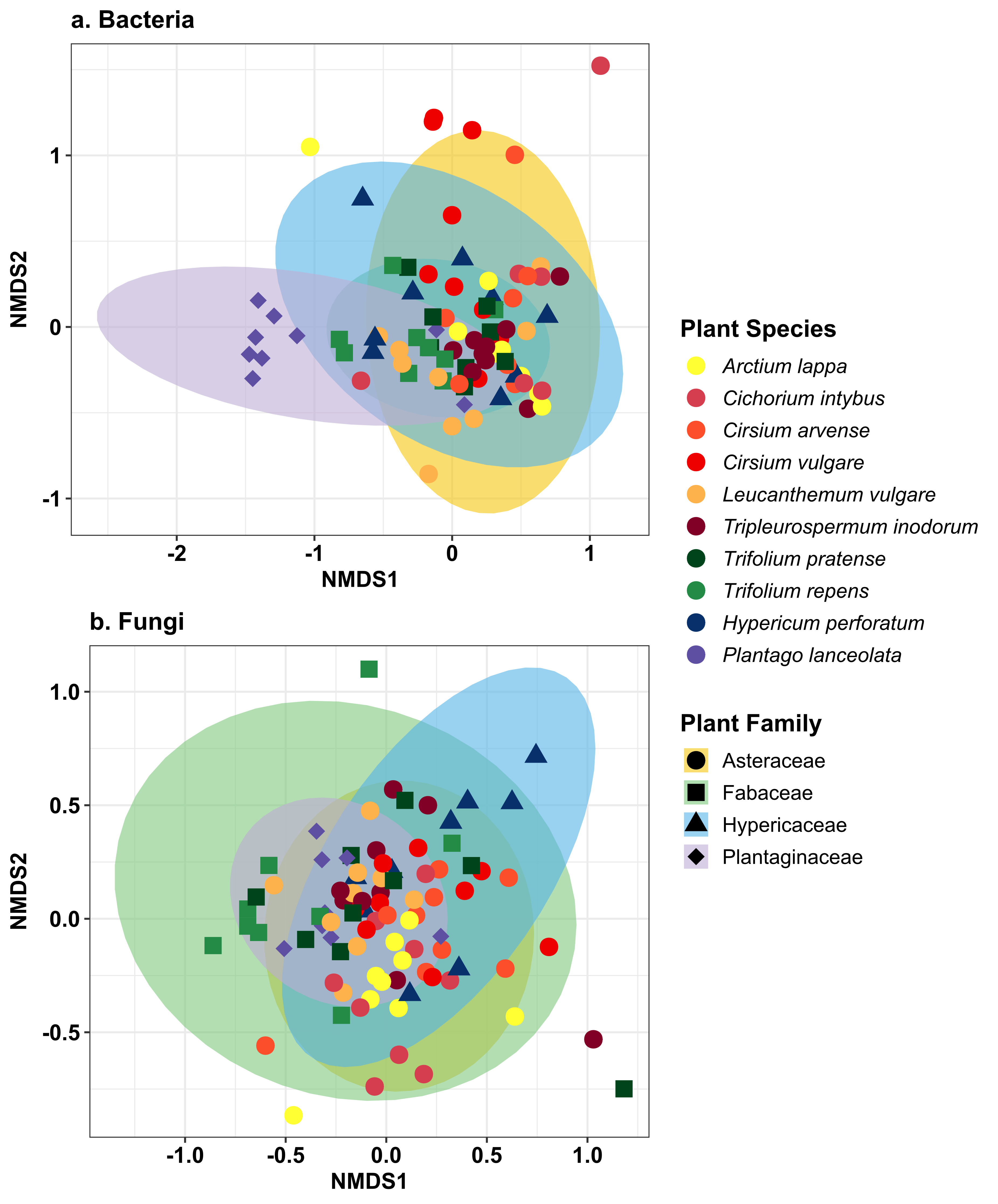
**

**Figure S2.** Non-metric dimensional scaling (NMDS) plots for (a) bacterial and (b) fungal families across plant species. Ellipses and shapes are coloured or shaped by plant family, respectively.


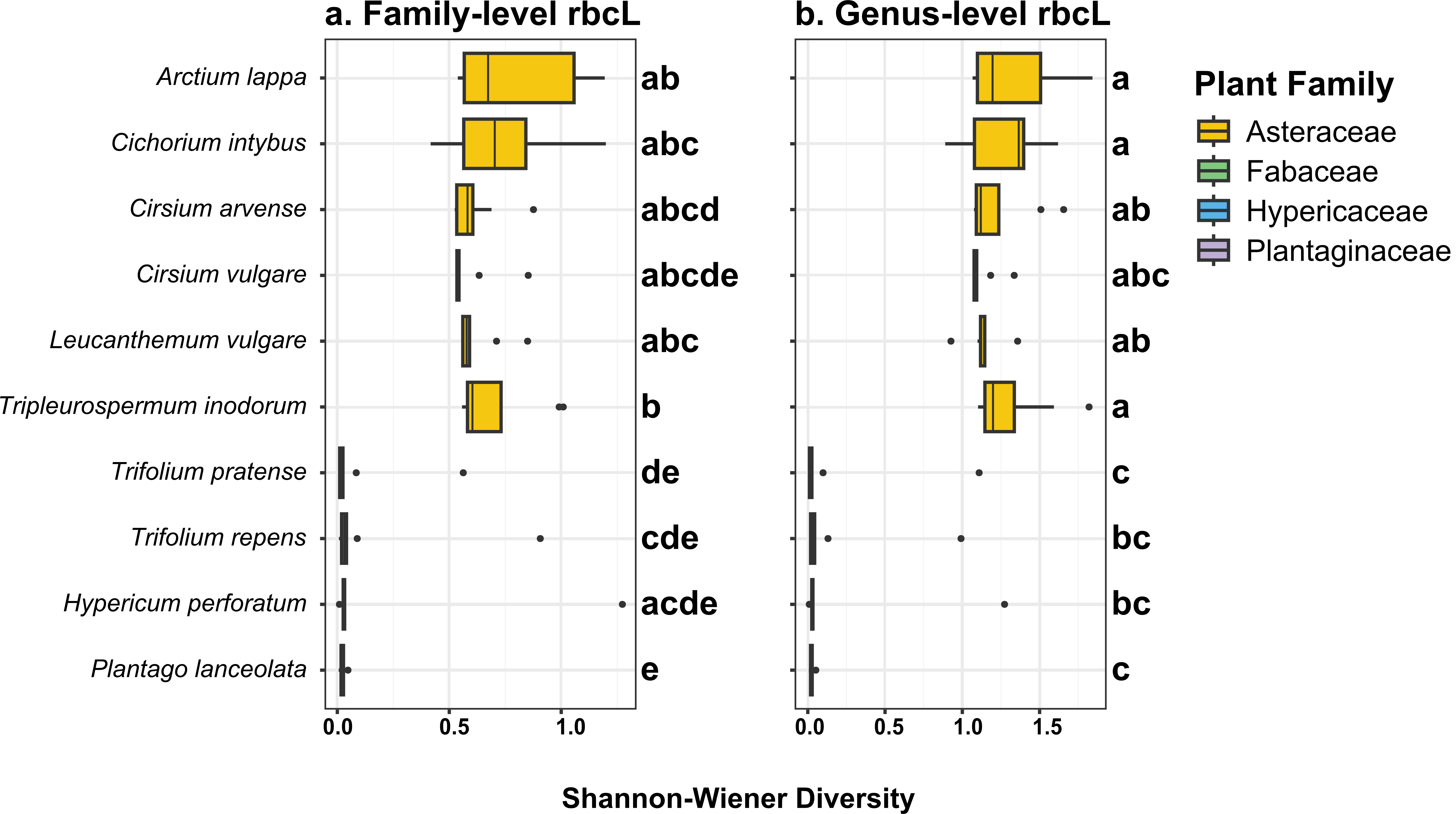


**Figure S3.** Shannon-Wiener alpha diversity across plant species for (a) plant family-level abundance data and (b) plant genus-level abundance data based on rbcL relative abundance. Boxplot bars are coloured by sample plant family. Significance letters based on post hoc Dunn’s test.


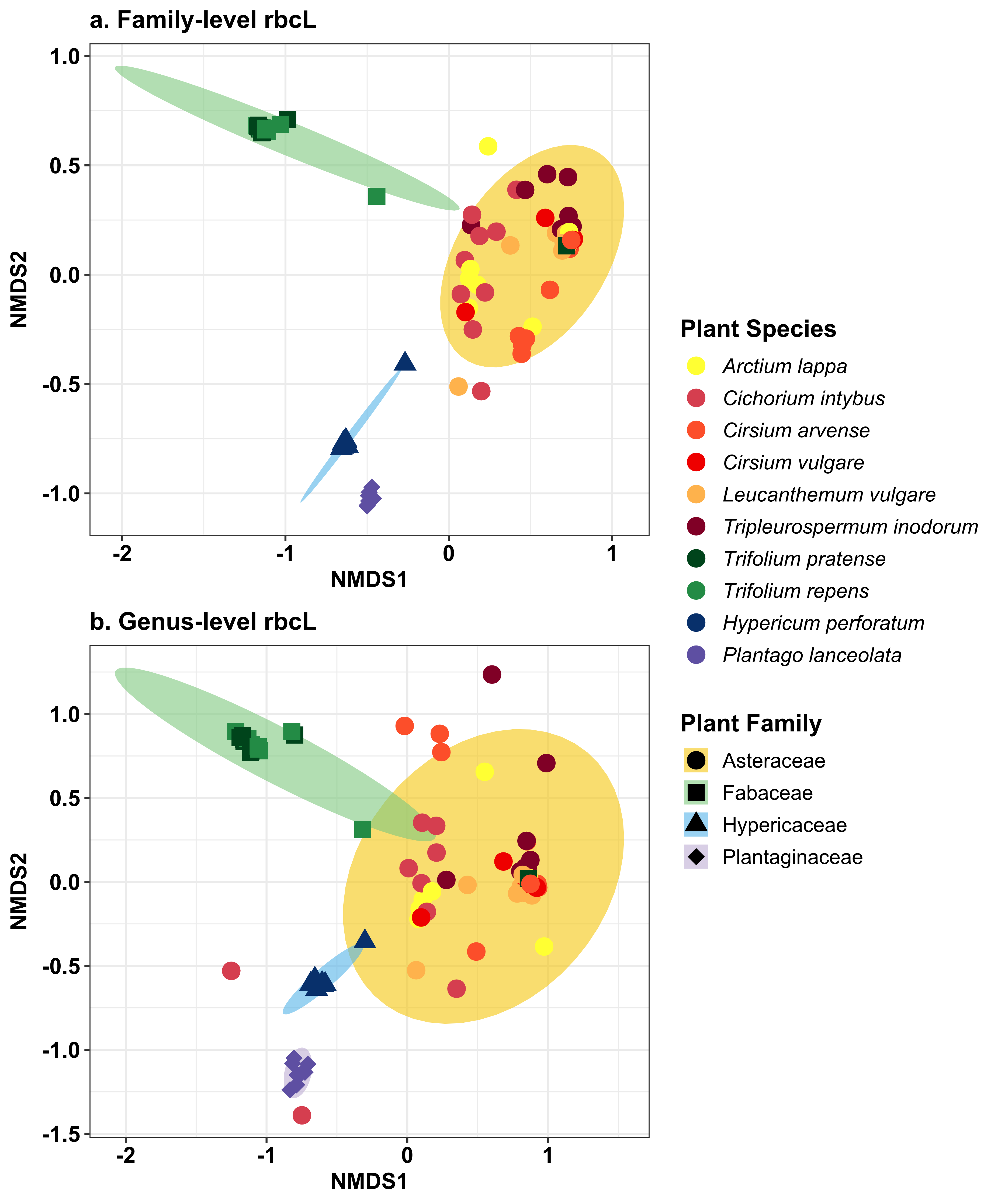


**Figure S4.** Non-metric dimensional scaling (NMDS) plots for (a) plant data at the family-level and (b) plant data at the genus-level across sample plant species. Ellipses and shapes are coloured or shaped by plant family, respectively.


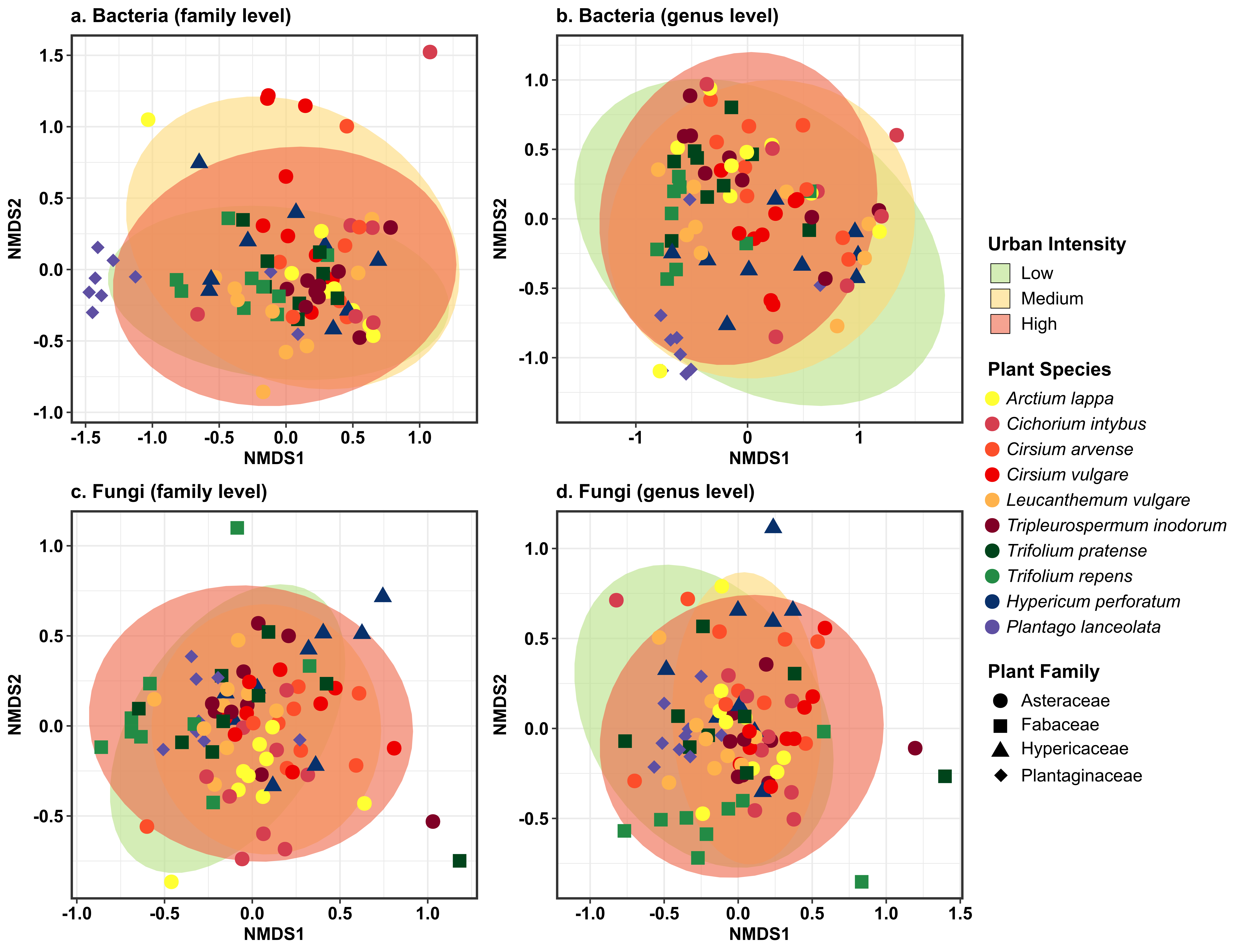


**Figure S5.** Non-metric dimensional scaling (NMDS) plots for (a) bacteria family level, (b) bacteria genus level, (c) fungi family level, and (d) fungi genus level across plant species. Ellipses are coloured by urban intensity and shapes represent plant family.
